# Examining the COVID-19 impact on cancer surgery in Ireland using three national data sources

**DOI:** 10.1016/j.gloepi.2024.100159

**Published:** 2024-08-03

**Authors:** Mengyang Zhang, Caitriona Kelly, Triona McCarthy, Paula Tierney, Aline Brennan, Louise Burke, Caitriona McGrath, Maeve Mullooly, Deirdre Murray, Kathleen Bennett

**Affiliations:** a School of Population Health, RCSI University of Medicine and Health Sciences, Dublin 2, Ireland; b National Cancer Control Programme, Dublin, Ireland; c National Cancer Registry Ireland, Cork, Ireland; d Department of Pathology, Cork University Hospital/School of Medicine, University College Cork, Cork, Ireland; e National Specialty Quality Improvement Programmes, Royal College of Physicians of Ireland, Dublin 2, Ireland; f School of Public Health, University College Cork, Cork, Ireland

**Keywords:** cancer surgery, COVID-19, Ireland, public-private healthcare system

## Abstract

**Background:**

The healthcare system in Ireland was profoundly affected by COVID-19. This study aimed to explore the impact of the pandemic on cancer surgery in Ireland, from 2019 to 2022 using three national health data sources.

**Methods:**

A repeated cross-sectional study design was used and included: (i) cancer resections from the National Histopathology Quality Improvement (NHQI) Programmes; (ii) cancer surgery from the National Cancer Registry Ireland (NCRI), and (iii) cancer surgery from Hospital Inpatient Enquiry (HIPE) System. Cancer surgery was presented by invasive/in situ and invasive only cancers (NCRI & HIPE), and by four main cancer types (breast, lung, colorectal & melanoma for NCRI & HIPE data only).

**Results:**

The annual number of cancer resections (NHQI) declined by 4.4% in 2020 but increased by 4% in 2021 compared with 2019. NCRI data indicated invasive/in-situ cancer surgery for the four main cancer types declined by 14% in 2020 and 5.1% in 2021, and by 12.3% and 7.3% for invasive cancer only, compared to 2019. Within HIPE for the same tumour types, invasive/in situ cancer surgery declined by 21.9% in 2020 and 9.9% in 2021 and by 20.8% and 9.6% for invasive cancer only. NHQI and HIPE data indicated an increase in the number of cancer surgeries performed in 2022.

**Conclusions:**

Cancer surgery declined in the initial pandemic waves suggests mitigation measures for cancer surgery, including utilising private hospitals for public patients, reduced the adverse impact on cancer surgery.

## Highlights

• Cancer surgery services were impacted by the COVID-19 pandemic in the initial three waves and recovered to pre-pandemic patterns by 2022.
• Data from the National Specialty Quality Improvement Programme and Hospital Inpatient Enquiry System provides continuous near real-time monitoring and assessment of cancer surgery.
• Findings supported the potential utilisation for near real-time monitoring and assessment of healthcare services during an unprecedented pandemic.
• The provision of surgical services in private hospitals for all patients substantially mitigated the impact on cancer surgery in the Irish setting.

## Introduction

The COVID-19 pandemic brought unprecedented challenges to healthcare systems worldwide. Cancer services were no exception, with disruptions experienced across the spectrum of care, from diagnosis to treatment including surgeries [1]. In the European Union (EU), 15% of countries reported more than 50% of their cancer care services were disrupted between October 2020 and February 2021, compared to pre-pandemic levels [2], with cancer surgeries most affected by delays and cancellations ahead of chemotherapy, immunotherapy and radiotherapy, according to a survey among European cancer registries [3].

Establishing COVID-19-free surgical pathways was important for ensuring adequate infection control precautions, however, this process was both time- and resource-consuming [4]. Many surgical departments had to contend with redeployment of resources, resulting in the cancellation or postponement of cancer surgeries. Modelling conducted across 190 United Nation (UN) member countries at the initial stage of the COVID-19 pandemic estimated that during the 12-week “peak COVID-19” period in 2020, 2,324,070 cancer surgeries (or 37.7% of expected cancer surgeries during this period) would be cancelled or postponed based on the estimates of global surgical data [5]. An international cohort study involving participants from 61 countries across high, middle, and low income settings found that one in seven cancer patients experienced delays in elective surgery due to COVID-19 pandemic mitigation measures [6].

High-quality data are critical for accurate assessment of cancer services, facilitating efficient planning and allocation of resources. Cancer registries are considered a high-quality data source, collecting and recording demographic and clinical data on all cases of cancer diagnosed [7]. However, to ensure accuracy and completeness of cancer registry data, a lengthy data quality process is required, limiting its use in near real-time. For timely analysis of patterns and changes in cancer surgery, complementary data sources with near real-time data, distinct from the conventional registry-based data, are often required for acute service planning.

This study aimed to explore the impact of the COVID-19 pandemic on cancer surgery procedures in Ireland, by comparing the period 2020–2022 with that of 2019 using data from National Cancer Registry Ireland (NCRI), the National Histopathology Quality Improvement (NHQI) programme and the Hospital In-Patient Inquiry (HIPE) system, across all cancers and by four cancer types.

## Methods

### Study design and setting

This study used a repeated cross-sectional design with data from three national health data sources in Ireland across five pandemic waves. COVID-19 pandemic waves were defined according to timelines outlined by the Health Protection Surveillance Centre [8] and included March 2020 – July 2020 (wave 1, non variant of concern), August 2020 – November 2020 (wave 2, non variant of concern), December 2020 – June 2021 (wave 3, alpha variant), July 2021 – December 2021 (wave 4, delta variant), and January 2022 – December 2022 (wave 5, omicron variant) [9,10].

The healthcare system in Ireland is a complex combination of public and private services, with many interdependencies and overlap of payers and providers. Private sector healthcare can help to improve the efficiency of and satisfaction with healthcare provision, although involvement of the private sector varies significantly across Europe under different universal health coverage and healthcare policies [11]. In Ireland, both sectors provide cancer treatments, including cancer surgery. Pre-pandemic, approximately 20% of selected cancer surgery took place in private hospitals, most often for common cancers (such as breast and lung cancer) [12], with most complex primary cancer surgery taking place in public hospitals.

The public healthcare system in Ireland is managed by the Health Service Executive (HSE) and is government funded. It is responsible for providing healthcare services to the population. Although everyone in Ireland is entitled to receive care in the public healthcare system, depending on a person's income, individuals may have to contribute a certain amount to emergency care and in-patient hospital admissions (€100 for attendance at an emergency department without a General Practitioner (GP) referral, and previous in-patient charges were €80 per night capped at €800 in a year but public in-patient charge was abolished for everyone in April 2023 [13]). Approximately one third of the population are entitled to free medical care. In the public hospital system, most cancer surgery is centralised to nine cancer centres and one satellite breast centre [14].

The private healthcare system in Ireland is accessed either by the ability to directly pay for the care or with private health insurance and is provided by a number of private hospital systems, which are distinct from public hospitals in terms of services provided and demographics of the patients they serve [15]. By the end of 2023, 7.6% of health insurance plans in Ireland were non-advanced plans, which only entitles patients to private accommodation in public hospitals [16].

During the initial response to the COVID-19 pandemic, an agreement between the HSE and private hospitals allowed for the provision of some essential public healthcare services in private hospitals to support the continued functioning of the public system that experienced a surge in demand amidst constrained capacity. This meant that some public patients received their cancer care and treatment (including surgery) in private hospitals during the pandemic [17]. Although this care was provided in private hospitals, it remained funded by the public system. This arrangement was similar to, but distinct from, the existing National Treatment Purchase Fund arrangement introduced to tackle waiting lists in the public sector by funding public patient treatment in private hospitals [18].

### Data sources

There are three main sources of available data on cancer surgery or resection in Ireland, including the National Histopathology Quality Improvement (NHQI) Programme, the National Cancer Registry Ireland (NCRI), and the Hospital Inpatient Enquiry (HIPE) System. The NHQI programme and HIPE provide near real-time data. Table 1 provides an overview of the data sources, features, and the indicators used as part of this analysis. Data from all three sources were ascertained.

**Table 1:** Overview of the three data sources and available indicators used in the study.

Data source	NHQI	NCRI	HIPE
Indicator	Cancer resection	Primary cancer surgery	Cancer surgery
Scope	All public and private laboratories in Ireland except one private laboratory	All Irish hospitals	All Irish public hospitals
Cancer	Invasive cancers, carcinoma in situ, and some specimens with no residual tumour*	Invasive cancers and carcinoma in situ (C00-C43, C45-C96, D00-D03, and D05-D09)	Invasive cancers and carcinoma in situ (C00-C43, C45-C96, D00-D03, and D05-D09)
Year	2019–2022	2019–2021	2019–2022
Characteristic	Near real-time data; population representative	Cancer registry data; population representative	Near real-time data; public healthcare system
	ICD codes not collected for NHQI dataset	2-year collection period	Activity for the pre-pandemic period
Analysis	Monthly number of cancer resections	Monthly number of cancer surgeries: both invasive and in situ, invasive only, four selected cancer types, and in public and private hospitals	Monthly number of cancer surgeries: both invasive and in situ, invasive only, and four selected cancer types

NHQI: National Histopathology Quality Improvement programme; NCRI: National Cancer Registry Ireland; HIPE: Hospital In-Patient Inquiry system. For NCRI and HIPE data, the principal diagnosis, coded as per the ICD-10-CM Diagnosis Code classification, followed by procedure group code, were used for data extraction. *Cancer resection may include a very small volume of cases that are not primary cancer resections.

The NHQI Programme was introduced by the Faculty of Pathology in the Royal College of Physicians of Ireland (RCPI) in collaboration with the National Cancer Control Programme in 2009. It is viewed as a near real-time source of pathology data collected from 21 public hospitals and seven participating private laboratories [19]. All laboratories in Ireland with the exception of one private laboratory provide data to the NHQI programme, meaning that the programme data are nationally representative. The NHQI programme aims to enhance quality of patient care and patient safety by providing evidence based, quality data on key performance indicators for pathology diagnostics. The NHQI programme records information on cancer resection specimens which may be different to the number of primary cancer surgeries performed as reported in NCRI data, due to the recording of all subsequent partial removal of tumour tissue as individual cases. A specific coding system is used for the classification of procedures. Data up to and inclusive of December 2022 were included; data completeness was 95% in 2019, 98% in 2020, 99% in 2021, and 99% in 2022.

The NCRI collects and records information on all cancer cases diagnosed in Ireland, in both public and private hospitals, since 1994 [7]. Because the NCRI is a population based cancer registry, and subject to a rigorous data quality process, the data available for inclusion were up to and inclusive of December 2021 and were extracted in April 2023.

The HIPE system, managed by the Healthcare Pricing Office (HPO) in the HSE, is the national database of in-patient and day-case health and social care occurring in public hospitals in Ireland. HIPE records demographic, clinical and administrative data on all discharges from, and deaths in, acute public hospitals nationally. The database is updated on a monthly basis [14], but only includes the public healthcare system. Data from private hospitals are not included in HIPE [20], which means that publicly-funded surgeries taking place in private hospitals during the COVID-19 pandemic were not recorded on HIPE. At present, there is no HIPE equivalent for private hospitals. For the purposes of this study, HIPE online data up to December 2022 inclusive were extracted on October 10th 2023.

### Cancer surgery definitions

For the NHQI data, the number of cancer resection specimens (internal code P03) was used as the indicator for cancer surgery. Within the NHQI programme, cancer resection is defined as “wide local excisions and excisions of entire organ(s), which may also include specimens with no residual primary tumour – post prior wide local excision, completion mastectomy/lobectomy, in situ/non-invasive disease or post neoadjuvant treatment, also included are local excisions/therapeutic excisions such as gastrointestinal polypectomy specimens/endoscopic mucosal resection – for excision of a polyp/cancer in the anus or oesophagus” [21]. In some instances, cancer is not detected after pathological examination, however these cases are not re-categorised leading to the inclusion of a very small volume of cases that are not primary cancer resections.

In the NHQI data, the monthly number of cancer resections refers to the number of cancer resection specimens from all participating laboratories, rather than the absolute number of cancer surgeries performed or the absolute number of patients undergoing cancer resections. For the purposes of this study, aggregated numbers of cancer resection specimens by month and year were obtained from the NHQI programme for all months from 2019 to 2022. Aggregate data stratified by cancer type were not available for inclusion nor was it possible to identify primary cancer resections, which is different from the definition of cancer surgeries for NCRI and HIPE data. As a reliable indicator of trends in cancer surgery using near real-time data from both public and private hospitals, cancer resection data from NHQI was compared with the HIPE data that also utilise near real-time data but only from public hospitals. Comparison also made to the NCRI data that includes both public and private hospitals but required a longer period for data collection.

For NCRI and HIPE data, the principal diagnosis, coded as per the ICD-10-CM Diagnosis Code classification, followed by procedure group code, were used for data extraction. Cancer surgery was examined for two groups: (i) including both invasive cancers and carcinoma in situ (excluding non-melanoma skin cancer (NMSC)) (C00-C43, C45-C96, D00-D03, and D05-D09) and (ii) including only invasive cancers (excluding NMSC) (C00-C43 and C45-C96). Four cancer types were selected for additional comparison: breast (C50), lung (C34), colorectal (C18, C19, and C20), and melanoma (C43). Procedure groups in the NCRI were defined using the 6th edition of the ICD10-AM/ACHI/ACS [12]. The coding system used in the HIPE system for 2019 data was based on the 8th edition of the ICD10-AM/ACHI/ACS [22] and the coding system for 2020–2022 data was based on the 10th edition [20]. Procedure codes from the 8th and 10th editions were reviewed and compared across coding versions to ensure the accuracy of cancer surgery procedures and the consistency of comparison over time.

Data extracted from the NCRI related to “primary cancer surgeries”, defined as the first tumour-directed (excisional/destructive) surgeries within one year of diagnosis [12]. The NCRI data shows the aggregated monthly number of primary cancer surgeries for 2019–2021. If a patient had more than one invasive cancer of similar sites and/or morphological subtype, only the surgery to remove the first invasive cancer of that site was counted. The data were also categorised by location of surgery (public or private hospital).

Within the HIPE data, the monthly numbers of surgical procedures conducted in acute public hospitals nationally for either invasive or in situ cancers, and for invasive cancers only, were extracted for all months from 2019 to 2022, and aggregated by the hospital admission month and year. Male and female patients in all age groups and all types of admission and discharge were included. The number of patients can be aggregated using the *E*-MRN (Electronic Medical Record Number) codes, a type of unique identifier assigned by a hospital to individual patients, through the online information system. The E-MRN codes are encrypted medical numbers and not available to researchers. The data may include patients with multiple admissions (and hence surgical procedures), so it may include more than “primary cancer surgeries”.

### Statistical analysis

The total annual number of cancer resections was presented for NHQI data, and for NCRI and HIPE data, categorised as: (i) both invasive and in situ cancer surgeries and (ii) invasive only cancer surgeries. Percentage changes between 2019 and 2020/2021 and between 2020 and 2021 were calculated. Negative percentage values indicate a decrease and positive percentages an increase over time.

To examine the variation during the pandemic period between 2019 and 2021, the monthly number of all indicators was presented by invasive and in situ combined, invasive only, by data source (NHQI, NCRI, and HIPE), and across the initial four pandemic waves. NHQI and HIPE data were used to describe cancer surgery trends during 2022.

Monthly numbers of cancer surgeries were also presented across four cancer types – breast, lung, colorectal, and melanoma – from 2019 to 2021 inclusive using NCRI and HIPE data. These cancer types were chosen as they are among the most common incident cancers in Ireland [23] and complete NCRI data were available from 2019 to 2021. The difference between data sources and between years were summarised. More details are shown in the Supplementary Appendix Tables 1 and 2.

In addition, monthly number of cancer surgeries by hospital sector (public or private) across the same four cancer types was examined using NCRI data, in order to examine the coordination of cancer surgery between public and private services during the pandemic period 2019–2021.

## Ethical approval

The analysis of cancer surgery data was conducted using anonymised aggregated data, which was provided by the NHQI, NCRI, and HIPE for the purpose of research analysis and ethical approval was not required.

## Results

### Patterns of cancer surgery across the pre-pandemic and pandemic periods

The annual numbers of cancer surgeries and percentage change from 2019 to 2021 by data source are shown in Table 2. All three data sources showed that cancer surgery activity was lower in 2020 compared with 2019, and increased in 2021 compared with 2020. Only the NHQI programme data showed cancer surgery activity was greater in 2021 than in 2019.

**Table 2:** Total number of cancer cases by data source for 2019–2021 and absolute and % change between 2019 and 2020/2021 and between 2020 and 2021.

Cancer surgery indicator	2019	2020	2021	Absolute diff. 2020 to 2019	% diff.	Absolute diff. 2021 to 2019	% diff.	Absolute diff. 2021 to 2020	% diff.
NHQI Cancer resection	16,881	16,141	17,553	−740	−4.4%	672	4.0%	1412	8.7%
NCRI invasive + in situ	14,893	12,815	14,140	−2078	−14.0%	−753	−5.1%	1325	10.3%
NCRI invasive only	11,677	10,245	10,829	−1432	−12.3%	−848	−7.3%	584	5.7%
HIPE invasive + in situ	13,848	10,825	12,503	−3023	−21.8%	−1345	−9.7%	1678	15.5%
HIPE invasive only	12,185	9662	11,035	−2523	−20.7%	−1150	−9.4%	1373	14.2%

NHQI - National Histopathology Quality Improvement Programme. NCRI - National Cancer Registry Ireland. HIPE - Hospital Inpatient Enquiry system. %diff. Refers to percentage difference between two years.

NHQI programme data showed the total number of cancer resection specimens was 4.4% lower in 2020 compared to 2019, however, the number of cancer resection specimens was 4.0% higher in 2021 compared with 2019.

The NCRI data showed decreases in primary cancer surgeries in 2020 of 14.0% (both invasive and in situ) and 12.3% (invasive only) compared to 2019, and decreases of 5.1% and 7.3% respectively in 2021 compared with 2019.

HIPE data showed the largest reduction in cancer surgeries, with a 21.8% reduction for both invasive and in situ cancer surgeries in 2020 and a 9.7% reduction in 2021 compared to 2019, similar reductions were observed for invasive only cancer surgeries.

**Fig. 1:**
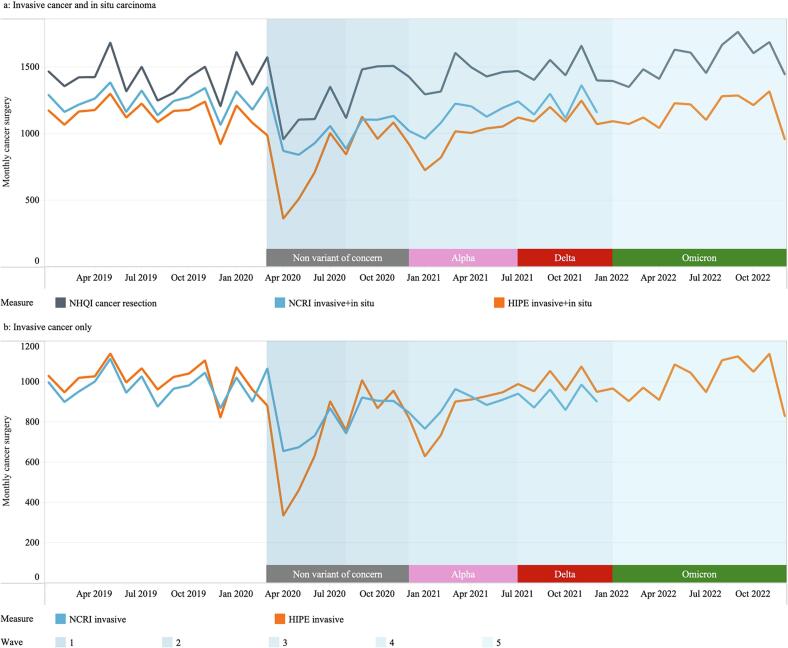
(a) Monthly trends from 2019 to 2022 in the number of cancer resections (NHQI) and cancer surgeries both invasive and in situ (NCRI and HIPE). (b) Monthly trends in the number of invasive cancers only within NCRI up to 2021 and HIPE up to 2022. NHQI - National Histopathology Quality Improvement programme. NCRI - National Cancer Registry Ireland. HIPE - Hospital Inpatient Enquiry system. NCRI data is from 2019 to 2021 only. NHQI – dark grey line, NCRI – blue line, HIPE – orange line. Virus variants by pandemic wave were non variant of concern in wave 1 and 2, Alpha in wave 3, Delta in wave 4, and Omicron in wave 5. (For interpretation of the references to colour in this figure legend, the reader is referred to the web version of this article.)

The patterns of cancer surgery between 2019 and 2022 by month and data source are shown in Fig. 1 (NCRI data was only available up to December 2021). Monthly numbers of both invasive and in situ surgeries and cancer resections are shown in Fig. 1a and the monthly surgeries for invasive cancers only, are shown in Fig. 1b.

All three data sources showed similar patterns of cancer surgery in 2019. NCRI and HIPE data recorded similar surgical activity which were lower than the number of cancer resections reported by the NHQI programme. The difference between NCRI and HIPE data widened at the beginning of the pandemic, with the largest differences found at the start of pandemic wave 1 (April 2020) and in wave 3 (December 2020 – January 2021).

Although the difference in monthly numbers of cancer surgeries and resections recorded in each data source increases after the start of the pandemic, each data source presents a similar pattern across the study period during the pandemic. For example, the numbers of cancer surgeries or resections recorded in each data source reduced in April 2020 compared with March 2020, but varied in the size of the reduction (NHQI reported numbers of resections reduced by 39.1%, the number of surgeries reduced by 35.4% in NCRI, and by 63.2% in HIPE; Fig. 1a). All three data sources also recorded decreases in surgical activity at the start of the second and third pandemic waves (starting August 2020 and December 2020) but returned to the average 2019 level from July 2021. There was no decline in surgical activity observed during wave 4 (starting July 2021).

The NHQI programme monthly cancer resections and numbers of invasive and in situ cancer surgeries using HIPE data for 2022 are shown in Fig. 1a. The monthly numbers of invasive cancer surgeries using HIPE data for 2022 are shown in Fig. 1b. In 2022, there was an increase in the number of cancer resections recorded by the NHQI programme compared to the average number recorded in 2019, and the number of invasive, and invasive and in situ cancer surgeries using the HIPE data had returned to the 2019 average level by 2022. The different data sources present a similar pattern over time.

### Patterns of cancer surgery according to four cancer types between 2019 and 2021

**Fig. 2:**
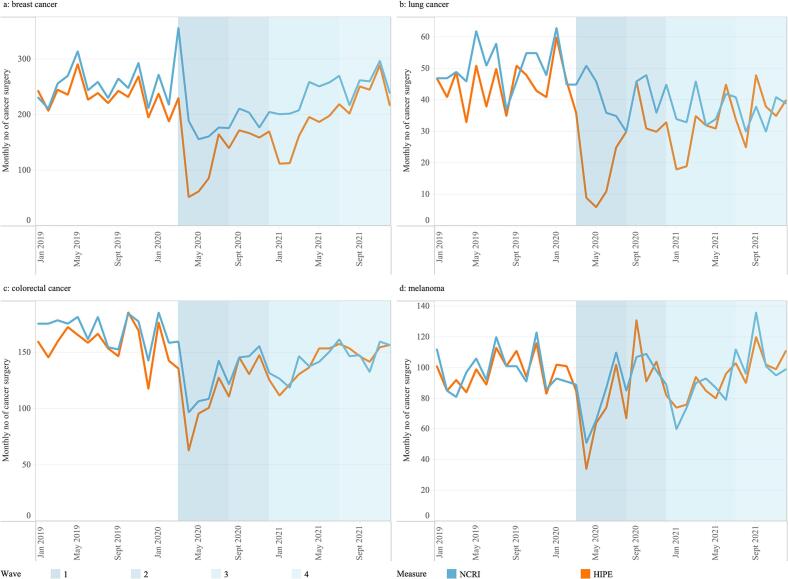
Trends in the number of cancer surgeries using NCRI and HIPE data for four cancer types including (a) breast cancer, (b) Lung cancer, (c) colorectal cancer and (d) melanoma for 2019–2021. Shading represents the waves 1–4 of the pandemic. Data are from the National Cancer Registry Ireland (NCRI) and Hospital Inpatient Enquiry (HIPE) System and include four cancer types: a-breast (C50), b-lung (C34), c-colorectal (C18, C19, and C20), and d-melanoma (C43). NCRI – blue line, HIPE – orange line. (For interpretation of the references to colour in this figure legend, the reader is referred to the web version of this article.)

Patterns of cancer surgery activity for breast, lung, colorectal and melanoma are presented in Fig. 2, using data from NCRI and HIPE. Details of annual differences and differences between data sources are shown in Appendix Table 1 and Table 2. During wave 1, differences were noted in cancer surgery activity recorded between NCRI and HIPE for breast (Fig. 2a) and lung cancer (Fig. 2b). For colorectal (Fig. 2c) and melanoma (Fig. 2d) similar trends for NCRI and HIPE were observed over the time period examined.

For breast cancer, the NCRI data reported a decrease of 17.5% between 2020 and 2019, with an even higher decrease of 22.3% in March and December between 2020 and 2019. There was an increase in annual surgery activity in 2021 compared with 2020 (16.9%). The HIPE data reported similar trends but with higher percentage differences. For lung cancer surgeries, the percentage annual difference between 2019 and 2020 were − 12.5% (NCRI) and − 31.1% (HIPE), with greater percentage differences identified when comparing just the March–December period (−17.6% (NCRI) and − 41.5% (HIPE)).

### Cancer surgery services between public and private sectors 2019–2021

**Fig. 3:**
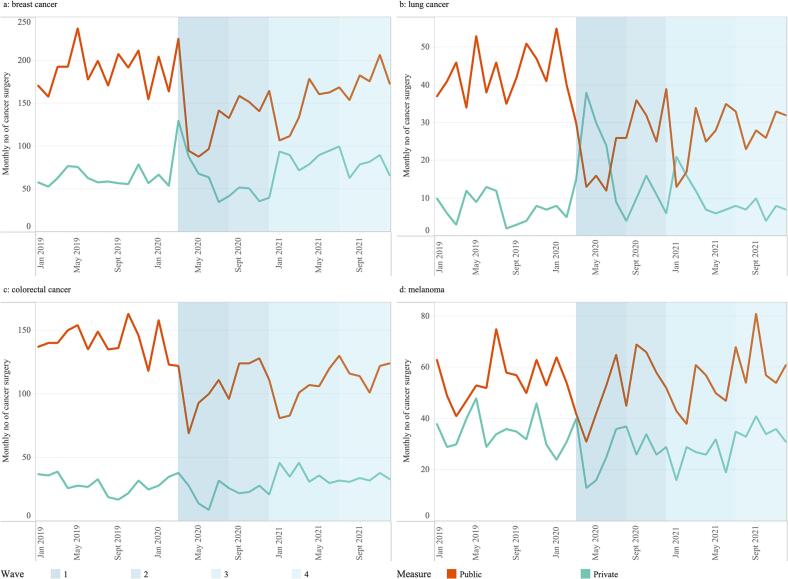
Trends in the numbers of cancer surgery within public and private hospitals, using NCRI data only, for four cancer types including (a) breast cancer, (b) Lung cancer, (c) colorectal cancer and (d) melanoma for 2019–2021. Shading represents the waves 1–4 of the pandemic. Data are from the National Cancer Registry Ireland (NCRI) and include four cancer types: breast cancer (C50), lung cancer (C34), colorectal cancer (C18, C19, and C20), and melanoma (C43). Public hospitals – brown line, Private hospital – teal line. The public hospitals include both public and other category, which contains surgeries in hospital types recorded as acute, cancer centre, maternity, Northern Ireland, overseas, UK, and radiotherapy. Private hospitals include private hospitals all over Ireland. (For interpretation of the references to colour in this figure legend, the reader is referred to the web version of this article.)

During the first wave of the pandemic, some publicly-funded activity moved into private hospitals, under arrangements to mitigate pandemic-related pressures in the public system. Patterns of surgery between the two sectors were examined using NCRI data (Fig. 3). The NHQI programme data includes both public and private data, but are not available for analysis separately. The NCRI data enables identification of data from both the public and private systems.

For breast (Fig. 3a) and lung cancer (Fig. 3b), there was a decline in cancer surgery activity within public hospitals but an increase in activity within private hospitals in waves 1 and 3. This pattern between public and private activity was not seen for colorectal cancer (Fig. 3c) or for melanoma (Fig. 3d). For melanoma, the variation in the monthly data was consistent across public and private hospitals, except August–September 2020 and February–March 2021.

## Discussion

Using data from three national healthcare data sources, this study examined cancer surgery activity during the first four of the five waves of the COVID-19 pandemic in Ireland. Compared with 2019, the total cancer surgery activity was lower in 2020 across all data sources examined. This is in keeping with international reports of cancer surgery cancellations and delays during the initial stages of the COVID-19 pandemic [1,5,6]. Elective surgical activity was reduced in order to maintain critical care capacity for COVID-19 patients and resources were reallocated to support the response to COVID-19, and maintain COVID-19-free surgical pathways [4]. In addition, some of the reduction in surgical activity is likely to reflect a decrease in cancer diagnoses as a result of a pause in screening services and a reduction in diagnostic capacities during this time [24].

Although a decrease in surgical activity was observed across all three data sources, HIPE (reporting on public hospital activity only) recorded a 21% decrease in 2020, whereas NCRI and the NHQI Programme data (including data on both public and private activity) recorded a 14% and a 4.4% decrease respectively compared to 2019 figures. As part of Ireland's COVID-19 management strategy to maintain key services, some publicly funded activity including surgery was transferred to private hospitals [17]. Therefore, part of the decrease reported by HIPE reflects a change in the location of surgery, suggesting that during the initial pandemic waves total cancer surgery activity was more accurately reflected in the NCRI and NHQI programme data – noting once again that NCRI data includes primary cancer surgery only, whilst NHQI data includes all cancer resections during this time.

The reduction in numbers of cancer surgeries and resections was particularly pronounced during the first and third waves, reflecting the infection control policies at the time [25] in response to high incidence of COVID-19 cases and high rates of COVID-19 hospitalisations [26]; the second wave differing with lower rates of COVID-19 hospitalisations and intensive care admissions, suggesting that non-COVID-19 related care could continue with less disruption during this period [26]. For a similar reason, HIPE and NCRI data sources showed the largest differences at the start of these waves. These were the times during the pandemic when the public hospital setting was most impacted by COVID-19 hospitalisations [26], with international evidence showing an association between increasing COVID-19 cases and reduced surgical capacity [6]. In Ireland, the arrangement that allowed public service surgeries to be performed in private hospitals was utilised during these periods of additional demands in order to maintain surgical services. This explains the decrease in HIPE numbers without an equivalent decrease in NCRI cancer surgery numbers. Similar strategies were adopted internationally. A review of health system response to the COVID-19 pandemic in Mediterranean countries highlighted that the utilisation of private sector capacity increased the provision of intensive care unit (ICU) beds and health professionals through public-private partnerships, which was crucial to meeting immediate needs during the first pandemic wave [27]. Further, modelling related to hospital capacity and demand during the pandemic period in the UK showed that utilising the private health sector, one of the successful interventions, increased available resources and health workforce to fulfil the demand for COVID-19 care [28].

For trends by specific cancer types the numbers reported for colorectal cancers and melanoma by HIPE and NCRI were found to be similar, suggesting fewer public patients underwent surgery for these cancer types in the private hospitals at the time of pandemic, and that decreases in the patterns represent reductions in the overall number of cancer surgeries performed. For colorectal cancers, endoscopy services in both public and private hospitals were impacted by infection prevention and control (IPC) concerns with neither sector performing routine numbers of diagnostic procedures, a finding replicated internationally [29], resulting in fewer colorectal diagnoses during this period compared to pre-pandemic levels, and with pausing of screening services also a factor. As to the pattern seen for melanoma, this requires further investigation and will be the subject of a future study.

On the other hand, decreases were observed in the number of lung cancer surgeries using the HIPE data, without the same corresponding decrease in NCRI data. Internationally, surgery types requiring more intense perioperative care (such as lung cancer) were associated with increased risk of cancellation [6], with many countries reporting major disruptions to lung cancer surgeries at the Global Forum of Cancer Surgery [30]. As many lung cancer patients require a predictable ICU stay post-operatively, a safety-netting decision was made in Ireland to move these public patients to the private hospitals for their surgeries, in order to reduce this risk of cancellation [17]. Therefore, the reduction in lung cancer surgery observed in the HIPE numbers is partially explained by a change in surgical location, rather than simply a reduction in the number of public surgeries.

For breast cancer, there was an increase in surgical activity in March 2020, likely due to surgeries being expedited in the period before cases of COVID-19 were reported in Ireland. The patterns for breast surgeries were lower in 2020 than in 2019, reflecting fewer breast cancers diagnosed during this period, likely influenced by the pause in screening services [31] and delays in histopathology [14]. Similar to lung cancer, there was a decrease in the number of breast cancer surgeries performed in public hospitals at the start of the first and third waves, with a corresponding increase in private activity. However, this was less pronounced, suggesting that fewer breast cancer surgeries moved from public to private hospitals. Breast cancer surgery activity was greatly impacted, largely driven by declines in breast cancer diagnoses due to the pauses in breast screening, whilst the use of private hospitals helped to mitigate against potentially significant reductions in lung cancer surgery activity.

Overall, the patterns of cancer surgeries from the different sources were similar, although the absolute numbers differed. All three data sources showed that cancer surgery services were impacted by the COVID-19 pandemic but recovered over time. The NHQI programme and HIPE may represent near real-time data sources that would be useful for monitoring trends in cancer surgery and allow for service planning, in advance of definitive data from the NCRI. However, the utility of HIPE data during the COVID-19 pandemic period was limited due to the relocation of some public surgeries to private hospitals during the early stages of the pandemic. Therefore, decreases in HIPE activity during this period are not a true reflection of the changes in overall surgical activity. Whilst HIPE may be a useful near real-time data source outside of an emergency scenario, caution should be applied if using it for service planning in such a scenario, particularly if similar arrangements between the public and private sectors are introduced again.

In keeping with international findings “reveal[ing] the fragility of elective cancer surgery to lockdowns” [6], this study has highlighted the need for further resilience in the public healthcare system in Ireland. This is a continued challenge, with increasing medical emergency hospitalisations compromising elective surgical activity [32], particularly during winter virus seasons. Addressing this challenge will require ongoing investment and operational planning to ensure adequate surgery capacity in order to manage public health crises whilst minimising disruption to elective care, including surgical services [6].

Strengths of this study include the examination of monthly trends in cancer surgery from three distinct national data sources. The NCRI data captures almost all cancers in Ireland and is a reliable source of data on primary cancer surgeries. Consistent patterns were seen across the three data sources. This suggests that NHQI and HIPE indicators may act as good proxies for overall activity, and thus may provide a timely representation of cancer surgery trends, useful for acute service planning. However, limitations include that this study was mainly descriptive in nature, and, particularly, the lack of historical variation of cancer surgery using pre-pandemic data. For some of the data sources used, there was a lack of diagnosis and treatment coding details. The cancer resection data from NHQI included a wider definition of cancer surgery, which may not be consistent with the selection for NCRI and HIPE data. In addition, it was not possible to separate public and private cancer resections within the NHQI data source. HIPE data only represents a subset of the population in relation to cancer activity and, therefore, may be biased. Further, this study focused on surgery volume and includes no detailed data (such as cancer stage distribution, patient outcome, duration of surgical delays, and comorbidities) to fully examine the long-term impact of the pandemic. It should also be noted that this paper chose to focus specifically on cancer surgeries, and other treatment modalities are outside the scope of this paper. However, the limited surgical capacity during early pandemic waves may have led to a shift from surgery to alternative treatment options (for example, increased use of bridging endocrine therapies for breast cancer) [33], therefore, a reduction in cancer surgeries should not necessarily be conflated with a reduction in cancer treatment; additional studies are investigating systematic cancer treatments during this period.

This study presents a comprehensive description of variations in cancer surgery patterns observed during the initial four pandemic waves, which provides valuable insights into cancer surgery amidst the challenges posed by the COVID-19 pandemic. Understanding the trends in cancer surgery during different phases of the pandemic would have been important for timely informed decision-making and resource allocation. This study highlights how multiple data sources can provide a near real-time insight into surgical activity for continuous monitoring and optimisation of resources, thus ensuring that cancer surgeries can be efficiently managed even in the face of unforeseen disruptions, like the COVID-19 pandemic.

## Conclusion

Cancer surgical activity reduced during the early stages of the pandemic, however, in Ireland, the provision of surgical services in private hospitals substantially mitigated this impact, and surgical activity has since recovered.

The three national data sources collecting information on cancer surgeries have all shown similar trends during this time period, suggesting that the NHQI programme and HIPE databases could be used as near real-time sources of data for acute service planning, with validation provided by routinely recorded national cancer registry data.

## Funding

This project is funded by the 10.13039/501100001593Irish Cancer Society, grant reference number CMP21BEMU. The opinions, findings and conclusions or recommendations expressed in this material are those of the authors and do not necessarily reflect the view of the Irish Cancer Society.

## Authorship statement

**Mengyang Zhang:** Writing – review & editing, Writing – original draft, Visualization, Validation, Methodology, Investigation, Data curation, Conceptualization. **Caitriona Kelly**: Writing – review & editing, Writing – original draft. **Triona McCarthy:** Writing – review & editing, Writing – original draft. **Paula Tierney:** Writing – review & editing, Writing – original draft, Visualization, Validation, Methodology, Formal analysis, Data curation, Conceptualization. **Aline Brennan:** Writing – review & editing, Writing – original draft, Conceptualization. **Louise Burke:** Writing – review & editing, Conceptualization. **Caitriona McGrath:** Writing – review & editing, Resources. **Maeve Mullooly:** Writing – review & editing, Writing – original draft, Supervision, Resources, Funding acquisition, Conceptualization. **Deirdre Murray:** Writing – review & editing, Writing – original draft, Supervision, Resources, Data curation, Conceptualization. **Kathleen Bennett:** Writing – review & editing, Writing – original draft, Supervision, Project administration, Funding acquisition, Data curation, Conceptualization.

## Declaration of competing interest

All authors have no conflict of interest. KB is in receipt of funding from IQVIA and Novartis for unrelated research projects.

## Data Availability

The dataset included in this study can be requested following formal application from the National Cancer Registry Ireland (NCRI), the National Histopathology Quality Improvement (NHQI) Programme, and Healthcare Pricing Office (HPO).

## Appendix A Supplementary data

Supplementary material. Image 1

## References

[1] Riera R., Bagattini Â.M., Pacheco R.L., Pachito D.V., Roitberg F., Ilbawi A. (2021). Delays and disruptions in Cancer health care due to COVID-19 pandemic: systematic review. JCO Global Oncology.

[2] van Ginneken E., Siciliani L., Reed S., Eriksen A., Tille F., Zapata T. (2022). Addressing backlogs and managing waiting lists during and beyond the COVID-19 pandemic. Eurohealth.

[3] Joint Research Centre (2023).

[4] Glasbey J.C., Nepogodiev D., Simoes J.F.F., Omar O., Li E., Venn M.L. (2021). Elective Cancer surgery in COVID-19–free surgical pathways during the SARS-CoV-2 pandemic: an international, multicenter, comparative cohort study. J Clin Oncol.

[5] COVIDSurg Collaborative. (2020). Elective surgery cancellations due to the COVID-19 pandemic: global predictive modelling to inform surgical recovery plans. Br J Surg.

[6] Glasbey J., Ademuyiwa A., Adisa A., AlAmeer E., Arnaud A.P., Ayasra F. (2021). Effect of COVID-19 pandemic lockdowns on planned cancer surgery for 15 tumour types in 61 countries: an international, prospective, cohort study. Lancet Oncol.

[7] National Cancer Registry Ireland (2020).

[8] Health Protection Surveillance Centre (2022).

[9] Health Protection Surveillance Centre (2021).

[10] Health Protection Surveillance Centre (2022).

[11] Montagu D. (2021). The provision of private healthcare services in European countries: recent data and lessons for universal health coverage in other settings. Front Public Health.

[12] National Cancer Registry Ireland (2019).

[13] Health Service Executive (2023).

[14] Faculty of Pathology Royal College of Physicians of Ireland, National Cancer Control Programme, National Histopathology Quality Improvement Programme, National GI Endoscopy Quality Improvement Programme, National Radiology Quality Improvement Programme, DATA-CAN tUsHDRHfC (2021).

[15] Murphy A., Bourke J., Turner B. (2020). A two-tiered public-private health system: who stays in (private) hospitals in Ireland?. Health Policy.

[16] Health Insurance Authority (2023). Health insurance in Ireland Market Report. https://www.hia.ie/sites/default/files/2024-04/hia-market-report_2023.pdf.

[17] Mercille J., Turner B., Lucey D.S. (2022). Ireland’s takeover of private hospitals during the COVID-19 pandemic. Health Econ Policy Law.

[18] National Treatment Purchase Fund (2024). About the NTPF. https://www.ntpf.ie/home/about.htm.

[19] Royal College of Physicians of Ireland, Faculty of Pathology. National Histopathology Quality Improvement Programme (2022).

[20] Healthcare Pricing Office (2022).

[21] (2021). Royal College of Physicians of Ireland, Faculty of Pathology. Guidelines for the implementation of the National Histopathology Quality Improvement Programme Version 7.0.

[22] Healthcare Pricing Office (2020).

[23] National Cancer Registry Ireland (2022).

[24] Tierney P., McDevitt J., Brennan A., Walsh P. (2023).

[25] Crowley P., Hughes A. (May 2021).

[26] Health Protection Surveillance Centre (2022).

[27] Waitzberg R., Hernández-Quevedo C., Bernal-Delgado E., Estupiñán-Romero F., Angulo-Pueyo E., Theodorou M. (2022). Early health system responses to the COVID-19 pandemic in Mediterranean countries: a tale of successes and challenges. Health Policy.

[28] McCabe R., Schmit N., Christen P., D’Aeth J.C., Løchen A., Rizmie D. (2020). Adapting hospital capacity to meet changing demands during the COVID-19 pandemic. BMC Med.

[29] Santoro G.A., Grossi U., Murad-Regadas S., Nunoo-Mensah J.W., Mellgren A., Di Tanna G.L. (2021). DElayed COloRectal cancer care during COVID-19 pandemic (DECOR-19): global perspective from an international survey. Surgery.

[30] Are C., Tyler D., Howe J., Olivares A., Nissan A., Zippel D. (2022). Global forum of cancer surgeons: cancer surgery during the COVID-19 pandemic: impact and lessons learned. Ann Surg Oncol.

[31] Li T., Nickel B., Ngo P., McFadden K., Brennan M., Marinovich M.L. (2023). A systematic review of the impact of the COVID-19 pandemic on breast cancer screening and diagnosis. The Breast.

[32] Robb W., O’sullivan M., Brannigan A., Bouchier-Hayes D. (2004). Are elective surgical operations cancelled due to increasing medical admissions?. Ir J Med Sci.

[33] Lohfeld L., Sharma M., Bennett D., Gavin A., Hawkins S.T., Irwin G. (2024). Impact of the COVID-19 pandemic on breast cancer patient pathways and outcomes in the United Kingdom and the Republic of Ireland–a scoping review. Br J Cancer.

