# Supplementary material for: Examining the COVID-19 impact on cancer surgery in Ireland using three national data sources
Source: Glob Epidemiol. 2024 Aug 3;8:100159. doi: 10.1016/j.gloepi.2024.100159 (PMC11375242; doi:10.1016/j.gloepi.2024.100159)
Supplement: Supplementary file 1 — Supplementary material. [file mmc1.pdf]

Appendix A: Supplementary data

Table 1. Absolute difference and % change between data sources for 2019-2021.

	Breast				Lung			
Year	NCRI	HIPE	Absolute difference between NCRI and HIPE (%)		NCRI	HIPE	Absolute difference between NCRI and HIPE (%)	
2019	3,033	2,848	185	(6·1%)	601	527	74	(12·3%)
2020	2,502	1,829	673	(26·9%)	526	363	163	(31·0%)
2021	2,924	2,391	533	(18·2%)	440	400	40	(9·1%)
	Colorectal				Melanoma			
Year	NCRI	HIPE	Absolute difference between NCRI and HIPE (%)		NCRI	HIPE	Absolute difference between NCRI and HIPE (%)	
2019	2,047	1,906	141	(6·9%)	1,195	1,168	27	(2·3%)
2020	1,664	1,506	158	(9·5%)	1,075	1,037	38	(3·5%)
2021	1,731	1,723	8	(0·5%)	1,122	1,130	-8	(-0·7%)

Table 2: The absolute difference from previous year (numbers and percentage) and absolute difference in March-December in 2020 and 2021 compared with 2019 for four cancer types for 2019-2021.

Data source	NCRI					HIPE				
Year	Annual total	Absolute difference from previous year	Difference (%)	Absolute difference in Mar-Dec from pr2019	Difference (%)	Annual total	Absolute difference from previous year	Difference (%)	Absolute difference in Mar-Dec from pr2019	Difference (%)
**Breast**										
2019	3,033					2,848				
2020	2,502	-531	-17·5%	-579	-22·3%	1,829	-1,019	-35·8%	-995	-41·5%
2021	2,924	422	16·9%	-70	-2·7%	2,391	562	30·7%	-232	-9·7%
**Lung**										
2019	601					527				
2020	526	-75	-12·5%	-89	-17·6%	363	-164	-31·1%	-182	-41·5%
2021	440	-86	-16·4%	-134	-26·4%	400	37	10·2%	-76	-17·3%
**Colorectal**										
2019	2,047					1,906				
2020	1,664	-383	-18·7%	-376	-22·2%	1,506	-400	-21·0%	-414	-25·9%
2021	1,731	67	4·0%	-210	-12·4%	1,723	217	14·4%	-111	-6·9%
**Melanoma**										
2019	1,195					1,168				
2020	1,075	-120	-10·0%	-107	-10·7%	1,037	-131	-11·2%	-148	-15·1%
2021	1,122	47	4·4%	-10	-1·0%	1,130	93	9·0%	-2	-0·2%
